# Difference in the alveolar bone remodeling between the adolescents and adults during upper incisor retraction: a retrospective study

**DOI:** 10.1038/s41598-022-12967-y

**Published:** 2022-06-01

**Authors:** Ya Zheng, Chenjing Zhu, Meng Zhu, Lang Lei

**Affiliations:** Department of Orthodontics, Nanjing Stomatological Hospital, Medical School of Nanjing University, 30 Zhongyang Road, Nanjing, 210008 Jiangsu China

**Keywords:** Dental diseases, Oral diseases

## Abstract

The purpose of this study was to compare the difference of alveolar bone remodeling between the adolescents and adults in the maxillary incisor area during retraction. This retrospective study included 72 female patients who needed moderate anchorage to correct the bimaxillary protrusion. Subjects were further divided into the minor group (n = 36, 11–16 years old) and adult group (n = 36, 18–35 years old). Digital lateral cephalography and cone beam CT scanning were taken in each patient before (T0) and after treatment (T1). Cephalometry was conducted to assess incisor retraction, while alveolar bone thickness (ABT), alveolar bone distance (ABD), and alveolar bone area (ABA) were detected to assess changes in the alveolar bone. No difference in the inclination of upper incisors was observed at both T0 and T1 between two groups. Changes in the alveolar bone showed a similar tendency with bone apposition on the labial side and bone resorption on the palatal side in both groups. Less increase in the labial ABT (T1–T0) and more decrease in the palatal ABT (T1–T0) was found in the adult group, leading to less total ABT in the adult group. Higher reduction in ABD (T1–T0) was found in the adult group. Moreover, more decrease in the ABA (T1–T0) was found in the adult group. Adult patients have less alveolar bone support after treatment when compared with young adolescents. Orthodontists should take the age into consideration to reduce the potential periodontal risks during the treatment planning.

## Introduction

Orthodontic tooth movement (OTM), a physiological adaptive response to mechanic strain, is accompanied by dynamic remodeling of periodontium. The bone-bending theory and pressure–tension theory have been proposed to explain the alveolar bone remodeling of OTM. As for the bone-bending theory, it partially explains the rapid tooth movement occurring at the extraction site and in pediatric patients, since the bone is less calcified and more flexible^1^. In the most acknowledged pressure–tension theory, which was proposed by Sandstedt, Oppenheim and Schwarz in the early twentieth century, bone absorption by the osteoclasts on the compression side and bone apposition by the osteoblasts on the tension side are proposed to explain the bone remodeling events^2^. All these theories cannot fully explain the persistent controversy, i.e. “through the bone” or “with the bone” remodeling pattern^3^.

OTM is not merely an adaptation of the periodontium to mechanic force. It is a consequence of a vast array of interacting biological parameters and mechanic variables. Multiple factors, including force magnitude, speed as well as distance of OTM, affect the alveolar bone remodeling and type of OTM, i.e., uncontrolled tipping, controlled tipping, and bodily movement^4^. One factor that has often been neglected is the age of the patient. Alveolar bone undergoes dynamic remodeling during growth with reduced elasticity and flexibility in adults^5^. In addition, compared to young subjects, adult patients have reduced progenitor cells, decreased vascular supply and fibroblast density, leading to reduced bone turnover rates^6^; remodeling of the periodontal tissues occurred earlier and more prominent in the young than older animals^7^. Age may significantly affect the biological behavior of osteoblasts, the major bone forming cells in the alveolar bone, with reduced cell proliferation and bone formation capability in the osteoblasts from elderly subjects^8^; osteoblasts from elderly individuals produce more pro-inflammatory cytokines to attract osteoclast precursors^9^. Furthermore, adult patients may have differed alveolar bone structures due to less growth and bone apposition in adult patients; skeletal bone mineral density, mandibular alveolar bone mass, and alveolar bone thickness decreased significantly with a follow-up of 5 years in adult females^10^; similarly, age also negatively impact the health of alveolar bone and increase the prevalence of alveolar defects^11^. Therefore, the age of the patients may be one of the key elements in determining the periodontal status after OTM.

Bimaxillary protrusion is a common malocclusion deformity in the East Asia. Incisor retraction is often required to reduce the protrusion of lips as well as incisors^12^. The retraction of the anterior teeth may improve facial appearance, whereas it might bring about the risk of alveolar bone resoption and gingival recession, posing a huge challenge to the patient's periodontal health^13–15^. Cone beam CT (CBCT) has been widely utilized to explore the changes in the alveolar bone during orthodontic treatment in patients with bimaxillary protrusion^16^, and it has been shown that despite a controversy of increased or unchanged buccal alveolar bone wall after massive retraction, a consistent resorption of palatal alveolar bone is observed in adult patients with bimaxillary protrusion^4,17–20^. Although one may take it for granted that less alveolar bone thickness loss can be observed in young subjects, alveolar bone remodeling in young subjects is currently less available.

An intact tooth is anchored to the alveolar socket by the periodontal membrane. The alveolar bone provides solid support for the teeth to withstand the occlusal force. It has been acknowledged that OTM may bring about potential side effects on the periodontal tissue, such as gingival recession, bone dehiscence and fenestration^17^. Orthodontic treatment in adult patients is surging in China due to the improved economic status and increased esthetic demand. Whether alveolar bone remodeling differed between adults and adolescents has not been reported. Such scarcity of information regarding the effects of age on alveolar bone remodeling may deflect our clinical decision. We hypothesized that adolescent patients will have differed alveolar bone response to mechanic force when compared to adults.

## Methods

### Patient selection

The study protocol was evaluated and approved by the local ethics committee (Nanjing Stomatological Hospital, Medical School of Nanjing University, NJSH-2021INL-032). Written consent was obtained from participants or their parents (for participants less than 16 years old). The study follows the principles of the Declaration of Helsinki. All participants read and signed an informed consent form before participating in the study. Additionally, this study follows the recommendations proposed by the CONSORT Statement.

CBCT data, cephalometric data and medical records are all from Nanjing Stomatological Hospital, School of Medicine, Nanjing University. All patients started treatment from January 1, 2018 to December 31, 2019, and ended before June 30, 2021.

The inclusion criteria of subjects are as follows: (1) Complete CBCT data before and after treatment; (2) Canines and first molars have a Class I relationship, the angle between the treatment anterior incisors is less than 124°, and the maxillary arch crowding is less than 4 mm; (3) Extraction of four maxillary and mandible premolars in the treatment plan; (4) Female patients; (5) the age of the minor group is between 11–16 years old, and the age of the adult group is between 18–35 years old; (6) moderate anchorage without temporary anchorage devices being placed. Patients with previous orthodontic treatment, cleft lip palate, impacted anterior teeth, congenital tooth loss except third molars, systemic diseases and compromised periodontium were excluded.

The sample size required for this study was estimated by G*Power 3.1.9.4 (Franz Faul, Universität). A total of 72 patients (36 in each group) were required to determine a significant difference in inclination of incisors with a significance level of 0.05 and a power of 0.8 using independent samples t tests.

### Treatment procedures

All patients were treated with active self-ligating brackets (Empower, American Orthodontic, USA). Alignment was achieved by sequential insertion of 0.014- and 0.018-in. nickel–titanium (NiTi) archwires, followed by levelling with 0.016 × 0.022- and 0.018 × 0.025-in. NiTi archwires, and space closure was finished with 0.018 × 0.025-in. stainless-steel (SS) archwires by en masse retraction and sliding mechanics. A retraction force of 100 g was utilized to finish space closure. No palatal arch, Nance pad and temporary anchorage devices were placed to help anchorage control. A digital lateral x-ray radiograph was taken to perform the cephalometric analysis. The cephalometric data were presented in Table 1.

**Table 1:** The cephalometric data of minors and adults before and after the orthodontic treatment.

Parameter	Minors							Adults						
	T0		T1		T0–T1		P value	T0		T1		T0–T1		P value
	Mean	SD	Mean	SD	Mean	SD		Mean	SD	Mean	SD	Mean	SD	
SNA(Angle)	80.35	2.46	79.51	3.39	0.84	2.51	0.08	83.75	2.45	82.96	2.56	0.79	2.21	0.06
SNB(Angle)	76.40	2.96	76.52	2.98	− 0.12	1.76	0.71	79.59	2.44	78.38	3.12	1.21	2.32	0.01
ANB(Angle)	3.95	2.09	3.01	2.41	0.94	1.71	0.01	4.16	1.76	4.58	1.94	− 0.42	1.45	0.12
U1-SN(Angle)	110.94	7.20	102.90	7.15	8.04	7.41	0.00	110.29	9.22	101.23	7.01	9.06	9.62	0.00
U1-NA(Angle)	30.60	6.85	23.38	6.80	7.22	7.18	0.00	26.54	9.04	18.27	6.91	8.27	9.73	0.00
U1-NA(mm)	7.04	2.70	4.25	2.36	2.79	3.27	0.00	6.92	4.78	2.04	2.43	4.88	4.27	0.00
MP-SN(Angle)	38.01	5.68	36.94	6.39	1.06	2.44	0.02	34.52	5.23	34.93	5.92	− 0.41	2.72	0.41
MP-FH(Angle)	32.40	5.47	31.62	6.36	0.78	2.52	0.10	28.86	4.62	29.11	5.55	− 0.25	2.27	0.54
L1-MP(Angle)	95.24	7.84	92.16	11.06	3.08	6.79	0.02	98.01	6.96	94.65	6.14	3.36	7.66	0.02
L1-NB(Angle)	29.63	5.52	25.62	7.96	4.02	6.79	0.00	32.13	7.02	27.96	5.20	4.17	7.57	0.00
L1-NB(mm)	6.81	2.35	5.07	2.18	1.74	2.38	0.00	7.69	3.83	5.54	1.97	2.15	4.13	0.01
U1-L1(Angle)	115.82	8.95	128.00	9.73	− 12.17	9.33	0.00	117.17	11.10	129.19	9.24	− 12.02	13.49	0.00

*SD* standard deviation, *T* paired t test, *W* Wilcoxon test.

### CBCT image processing and measurements

**Figure 1:**
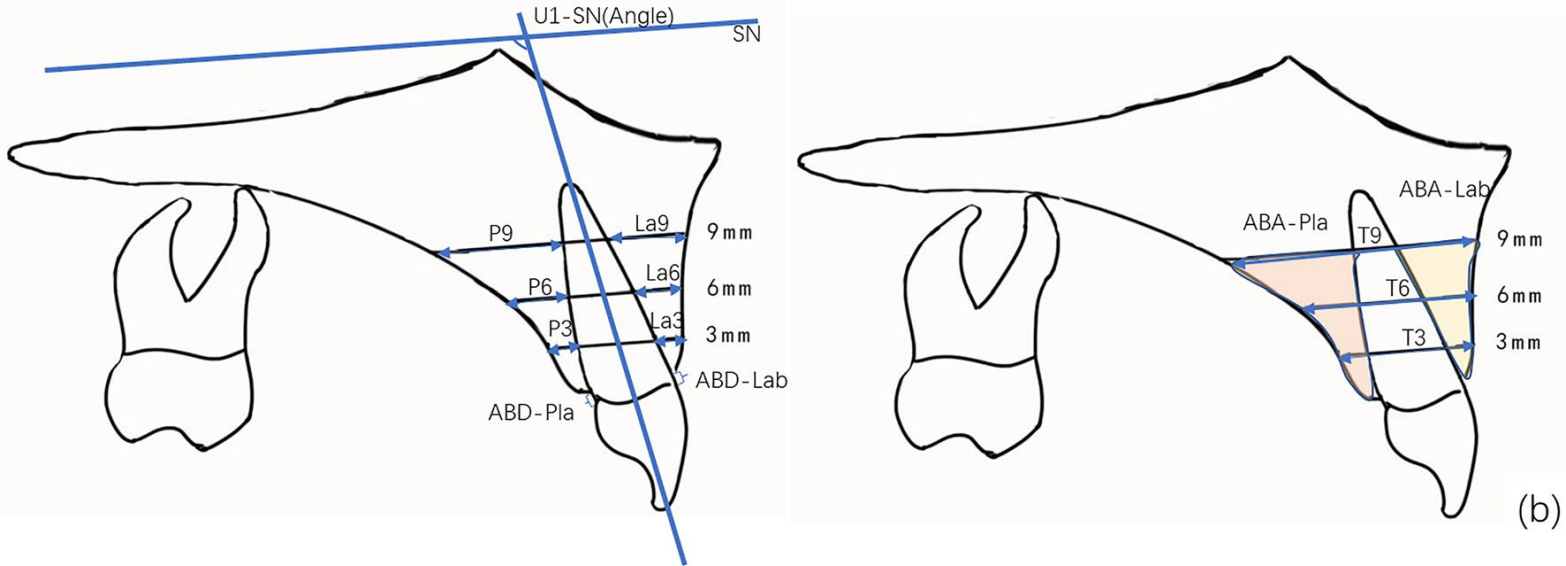
Schematic diagram of the CBCT measurement image, measurement mark points and measurement indicators. (**a**) CBCT reconstruction sagittal localization map (**b**) Schematic diagram of alveolar bone thickness, distance and area measurement. Along the long axis of the tooth, horizontal lines parallel to the SN plane were drawn at 3, 6 and 9 mm away from the root of the cementoenamel junction (CEJ), which was designated as crestal (La3 and P3), mid-root (La6 and P6) and apical third (La9 and P9); labial and palatal alveolar bone area (ABA-Lab and ABA-Pla); labial and palatal alveolar bone distance (ABD-Lab and ABD-Pla), defined as the distance from the CEJ to the alveolar ridge crest.

CBCT was taken with the same machine before and after treatment for all patients. All pretreatment and posttreatment CBCT were taken by the same machine. CBCT scans (NewTomVG, Quantitative Radiology, Verona, Italy) were taken before (T0) and after treatment (T1). The following imaging acquisition parameters were used: 16 × 16 cm field of view (FOV), 5 mA, 110 kV, and 3.6 s exposure time, which generated an isotropic voxel size of 0.3 mm. The effective dose of radiation was approximately 80 μSv. During the shooting process, the sagittal plane of the patient's head was perpendicular to the ground, and the FH plane was parallel to the horizontal plane. All data were reconstructed with NNT software of CBCT machine, and the sagittal longitudinal section was adjusted to pass through the root tip and parallel to the long axis of a single incisor. The reconstructed images were imported into Image J 2.0 software. Along the long axis of the tooth, horizontal lines parallel to the FH plane were drawn at 3, 6 and 9 mm away from the root of the cementoenamel junction (CEJ), representing the levels of the crestal, middle and apical third respectively. The following variables were measured (Fig. 1a,b):

1. alveolar bone thickness (ABT) on the buccal and palatal side at 3, 6 and 9 mm from the cementoenamel junction (CEJ), which was designated as crestal (La3 and P3), mid-root (La6 and P6) and apical third (La9 and P9);
2. labial and palatal alveolar bone area (ABA): the labial ABA refers to the area that was encircled by the alveolar ridge crest, labial alveolar bone surface, the 9 mm parallel line and the buccal side of the root, while the palatal ABA refers to the area that was enclosed by the alveolar ridge crest, palatal alveolar bone surface, the 9 mm parallel line on the labial and the palatal sides of the root;
3. labial and palatal alveolar bone distance (ABD), defined as the distance from the CEJ to the alveolar ridge crest.

### Statistical analysis

All measurements were conducted by one trained examiner. To reduce the measurement error, we took the average value of three measurements whose time interval was one-month as the result. Statistical analysis was performed with SPSS (version 23.0) for Windows (SPSS Inc, Chicago, Ill). The inter-examiner agreement was performed by another experienced investigator. Repeated measurements were examined by the paired t test (systematic errors) and the Dahlberg formula (casual errors)^21^. No significant systematic errors were found (P > 0.1), and the random errors were small, showing high rates of reproducibility. Demographics and clinical characteristics of the samples with respect to treatment duration was examined by independent sample t test or chi-square test. Intergroup comparisons before and after treatment were calculated, and if normally distributed, these were compared using paired t tests; if this was not the case, the Wilcoxon test was used. The significance level was set at 0.05 for all tests.

### Ethics approval and consent to participate

The study protocol was evaluated and approved by the local ethics committee (Nanjing Stomatological Hospital, Medical School of Nanjing University, NJSH-2021INL-032). Written consent was obtained from participants or their parents (for participants less than 16 years old).

### Approval for human experiments

The study protocol was evaluated and approved by the local ethics committee (Nanjing Stomatological Hospital, Medical School of Nanjing University, NJSH-2021INL-032). Written consent was obtained from participants or their parents (for participants less than 16 years old). The study follows the principles of the Declaration of Helsinki. All participants read and signed an informed consent form before participating in the study. Additionally, this study follows the recommendations proposed by the CONSORT Statement.

1. We identify the institutional and/or licensing committee that approved the experiments, including any relevant details.
2. We confirm that all experiments were performed in accordance with relevant named guidelines and regulations.
3. We confirm that informed consent was obtained from all participants and/or their legal guardians.

## Results

### Demographic and clinical characteristics

This study included data from 72 female patients (36 minors aged 11–16 and 36 adults aged 18–35). The average duration of the OTM of the adult group was 24.91 months, which was longer than that of the minor group (20.14 months), and the difference was statistically significant (P < 0.05).

Regarding the skeletal and dental parameters before treatment, no difference in the sagittal jaw pattern (ANB angle) and upper incisor position (U1-SN and U1-NA angle as well as distance) was observed between the minor and adult group, whereas a higher mandibular plane and less proclined lower incisor (L1-MP and L1-NB angle) was observed in the minor group(P < 0.05). In terms of the skeletal parameters after treatment, reduction in the mandibular plane and increase in the SNB was found in the minor group, leading to a reduced ANB angle; by contrast, no change in the mandibular plane and ANB angle was observed in the adult group (Table 1).

### Incisor retraction

Significant retraction of upper and lower incisors was observed in both the minor and adult group. The inclination of the upper incisor (U1-SN degree) in the minor group was (110.94 ± 7.20) ° and (102.90 ± 7.15)° before and after treatment, respectively, which was similar to the adult group, (110.29 ± 9.22)° and (101.23 ± 7.01)° before and after treatment, respectively. No difference was observed in the change of U1-SN(°) (T0–T1) with a reduction of 8.04 ± 7.41 and 9.06 ± 9.62 in the minor and adult group, respectively. Similar changes were also observed in the U1-NA(°) and U1-NA (mm), suggesting similar retraction of incisors in both groups. Despite the more proclined lower incisor(L1-MP degree) in the adult group (98.01 ± 6.96)° than the minor group (95.24 ± 7.84)° (P < 0.05), similar retraction was found in the minor (3.08 ± 6.79)° and adult group (3.36 ± 7.66)° after treatment. Similar changes in L1-NB(°) and L1-NB (mm) were observed in both group during treatment(Table 1).

### Alveolar bone thickness (ABT)

First, we examined the changes in the labial, palatal, and total ABT at the crestal, mid-root and apical third at the central incisor. Before orthodontic treatment (T0), less ABT on the labial side was found at the 3 and 9 mm in the adult than the minor group, while no difference was observed at 6 mm level; on the palatal side, no difference was found at both 6- and 9-mm level in both groups. After orthodontic treatment (T1), less bone support was consistently observed in the adults at all levels on the palatal side. Despite an increase in ABT on the labial side (T1–T0) in both groups, less increase in ABT was observed in the adults. Moreover, larger reduction in the palatal and total ABT (T1–T0) was found in the adult group, showing more bone loss on the palatal side in the adults (Table 2).

**Table 2:** Changes of the alveolar bone thickness at the crestal, mid-root and apical third in the central incisor.

Parameter	Minors							Adults									
	T0		T1		T1–T0		*P*^*a*^	T0		T1		T1–T0		*P*^*b*^	*P*^*c*^	*P*^*d*^	*P*^*e*^
	Mean	SD	Mean	SD	Mean	SD		Mean	SD	Mean	SD	Mean	SD				
La3(mm)	0.60	0.44	0.97	0.45	0.38	0.47	0.00	0.40	0.46	0.57	0.51	0.16	0.44	0.00	0.01	0.00	0.01
La6(mm)	1.18	0.33	1.47	0.52	0.29	0.42	0.00	1.19	0.44	1.28	0.49	0.09	0.46	0.12	0.87	0.04	0.02
La9(mm)	1.49	0.46	1.81	0.82	0.32	0.70	0.00	1.20	0.55	1.46	0.76	0.26	0.85	0.02	0.00	0.02	0.68
P3(mm)	3.20	1.18	2.32	1.55	− 0.88	1.21	0.00	2.58	0.89	1.10	0.85	− 1.48	0.79	0.00	0.00	0.00	0.00
P6(mm)	4.87	1.83	4.05	2.32	− 0.82	1.82	0.00	4.40	1.31	2.71	1.61	− 1.70	1.41	0.00	0.12	0.00	0.00
P9(mm)	6.77	2.46	6.44	3.12	− 0.33	2.25	0.24	6.88	1.93	5.29	2.56	− 1.59	2.67	0.00	0.79	0.04	0.00
T3(mm)	10.32	1.18	9.57	2.14	− 0.75	1.72	0.00	9.31	1.18	7.97	1.05	− 1.34	0.99	0.00	0.00	0.00	0.02
T6(mm)	11.41	1.78	10.58	2.23	− 0.84	1.72	0.00	10.70	1.58	9.12	1.46	− 1.58	1.47	0.00	0.03	0.00	0.01
T9(mm)	11.79	2.17	11.52	2.51	− 0.26	2.02	0.29	11.58	2.03	10.48	2.14	− 1.11	2.23	0.00	0.61	0.02	0.03

*SD* standard deviation, *T0* before orthodontic treatment, *T1* after orthodontic treatment, *T1–T0* difference value during T0 to T1.

^a^Represents the P-value of t test of T1–T0 in the minors group.

^b^Represents the P-value of t test of T1–T0 in the adults group.

^c^Represents the P-value of t test of T0 between the two groups.

^d^Represents the P-value of t test of T1 between the two groups.

^e^Represents the P-value of t test of T1–T0 between the two groups.

Next, we assessed changes at the lateral incisors. Regarding ABT before the treatment (T0), less ABT at all levels on the labial side was observed in the adults, while no difference was found in the palatal and total ABT between the two groups. In terms of ABT after the treatment (T1), consistent less ABT was found at all sites except at the La6 in the adults. For the changes in ABT (T1–T0), similar to the central incisors, less increase in the labial ABT and more decrease in the palatal ABT were observed (Table 3).

**Table 3:** Changes of alveolar bone thickness at the crestal, mid-root and apical third in the lateral incisors.

Parameter	Minors							Adults									
	T0		T1		T1–T0		*P*^*a*^	T0		T1		T1–T0		*P*^*b*^	*P*^*c*^	*P*^*d*^	*P*^*e*^
	Mean	SD	Mean	SD	Mean	SD		Mean	SD	Mean	SD	Mean	SD				
La3(mm)	0.65	0.45	0.71	0.48	0.06	0.51	0.34	0.45	0.50	0.33	0.42	− 0.11	0.49	0.05	0.01	0.00	0.04
La6(mm)	0.80	0.39	1.02	0.46	0.22	0.53	0.00	1.03	0.41	1.04	0.52	0.02	0.49	0.87	0.01	0.99	0.04
La9(mm)	0.92	0.60	1.37	0.67	0.47	0.66	0.00	0.70	0.46	0.94	0.65	0.20	0.48	0.00	0.02	0.00	0.01
P3(mm)	2.18	0.79	1.31	0.87	− 0.87	0.81	0.00	1.78	0.70	0.59	0.64	− 1.19	0.81	0.00	0.01	0.00	0.01
P6(mm)	3.60	1.27	2.51	1.51	− 1.08	1.03	0.00	3.19	1.12	1.65	1.38	− 1.55	1.34	0.00	0.07	0.00	0.03
P9(mm)	5.15	1.68	4.26	2.21	− 0.89	1.44	0.00	4.96	1.50	3.33	2.08	− 1.63	1.69	0.00	0.52	0.03	0.01
T3(mm)	9.13	1.03	8.17	1.19	− 0.88	0.94	0.00	8.37	1.05	7.11	0.77	− 1.26	0.88	0.00	0.00	0.00	0.02
T6(mm)	10.24	1.44	9.07	1.64	− 1.17	1.26	0.00	9.84	1.40	8.14	1.26	− 1.70	1.51	0.00	0.15	0.00	0.04
T9(mm)	10.45	1.74	9.58	2.09	− 0.86	1.24	0.00	9.93	1.49	8.48	1.94	− 1.44	1.59	0.00	0.07	0.00	0.03

*SD* standard deviation, *T0* before orthodontic treatment, *T1* after orthodontic treatment, *T1–T0* difference value during T0 to T1.

^a^Represents the P-value of t test of T1–T0 in the minors group.

^b^Represents the P-value of t test of T1–T0 in the adults group.

^c^Represents the P-value of t test of T0 between the two groups.

^d^Represents the P-value of t test of T1 between the two groups.

^e^Represents the P-value of t test of T1–T0 between the two groups.

### Alveolar bone distance (ABD)

**Figure 2:**
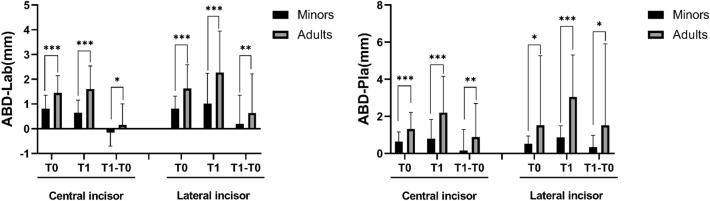
Changes in the labial and palatal alveolar bone distance in the central and lateral incisors before (T0) and after treatment (T1). *, *P* < 0.05; **: *P* < 0.01, ***, *P* < 0.001.

In terms of the ABD, which was measured from CEJ to alveolar bone crest, higher ABD was observed in the adults than the minors at T0 and T1 on both the labial and palatal side for both the central and lateral incisors. No significant difference in the changes of ABD (T1–T0) on the palatal side of central incisors and on the buccal side of the lateral incisor was observed in the minor group, whereas labial ABD (T1–T0) of the central incisor and the palatal ABD (T1–T0) of lateral incisor was increased in the minors; however, consistent increase in the ABD (T1–T0) was observed in the adults for all sites. In terms of comparison between the minor and adult group, larger increase in the changes of ABD (T1–T0) was observed in the adult group on both sides for both the central and lateral, indicating a possible bone loss in the adults during orthodontic treatment (Fig. 2).

### Alveolar bone area (ABA)

**Figure 3:**
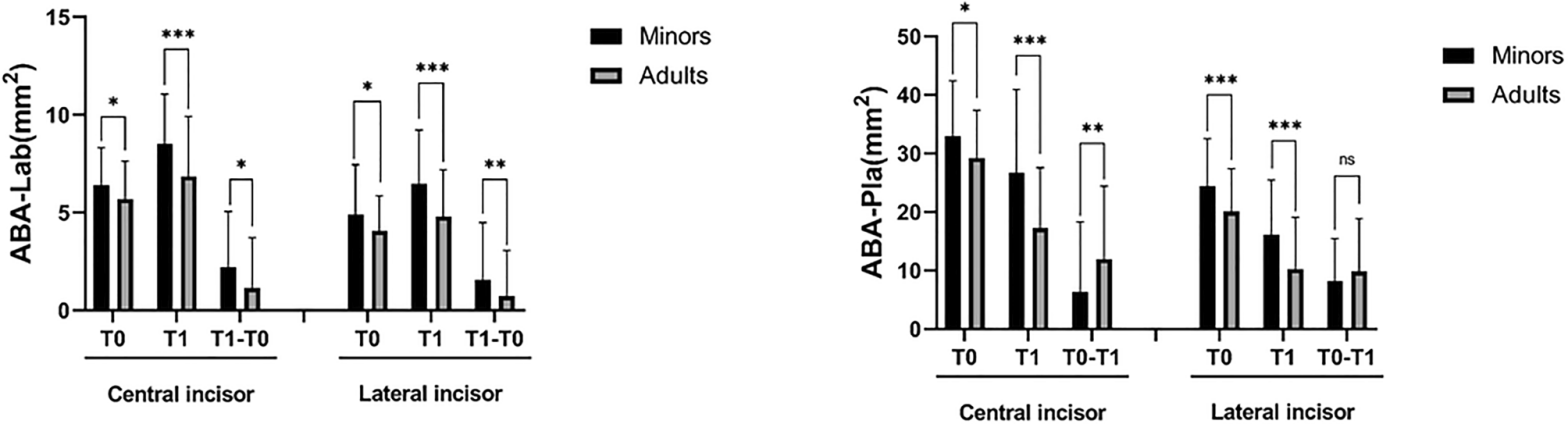
Changes in the labial and palatal alveolar bone area in central incisors and lateral incisors before (T0) and after treatment (T1). *, *P* < 0.05; **: *P* < 0.01, ***, *P* < 0.001.

Less ABA was observed in the adult group before (T0) and after (T1) treatment for both the central and lateral incisors than in the minor group. Regarding changes of ABA (T1–T0), more reduction on the palatal side and less increase on the labial side was observed in the adults, which further indicated less bone formation on the labial side and more bone resorption on the palatal side (Fig. 3).

## Discussion

OTM in adolescents and adults displays multiple differences, such as less root resorption in maxillary incisors of adolescents^22^, faster OTM in teenagers^6,23,24^. Orthodontists are often warned that the anatomic cortical plate on the buccal and palatal side constitute the limit of OTM, i.e. OTM should not be planned to be out of this bony wall^25^. Our present work further demonstrated that retraction of protrusive incisors in adolescents showed better alveolar bone support on both the palatal and buccal side. Periodontal adverse effects, such as gingival recession, bone fenestration, and dehiscence, are of great concern for orthodontist^26^. Our findings would significantly impact our decision on the timing and planning of orthodontic treatment from the perspective of periodontal health.

Orthodontists are often puzzled by a wide range of clinical response to similar mechanical force. The fast OTM in young subjects cannot fully be explained by the classical bone formation-resorption theory, whereas it might be partially explained by the bone-bending theory^27^. Indeed, bending at the alveolar crest occurs with forces within the range used by conventional orthodontic appliances, which may amount to a deflection of 35 μm at the initial stage of force application^27^. Pediatric bone displays a lower modulus of elasticity, bending strength, as well as lower bone density, all of which contribute to bending tendency of the alveolar bone under mechanical stress^28^. In our present study, less ABA was observed on the compression palatal side in adult patients, indicating that the bone apposition-resorption theory better explains the OTM in adults, while the mechanic bone-bending theory may partially participate in the OTM in adolescents.

Highly integrated cellular signaling events orchestrate the bone formation, absorption and OTM in the periodontal niche. Alveolar bone remodeling is dynamically regulated by bone-forming osteoblasts and bone-absorbing osteoclasts^28^. The osteoblasts and stromal stem cells express receptor activator of NF-κB ligand (RANKL), which binds to its receptor, RANK, on the surface of osteoclasts and their precursors to promote bone resorption; in addition, osteoblasts secrete osteoprotegerin, which binds to RANKL and protects the skeleton from excessive bone resorption^29^. Compared to elderly subjects, alveolar osteoblasts from young human subjects showed less proliferation capacity and lower bone formation capability^8^. Human osteoblasts from aged and young subjects respond similarly to short-term stimulation of proliferation and differentiation, while osteoblasts from elderly individuals express more pro-inflammatory interleukin-6, which may promote chemotaxis of osteoclast precursors^9^. Interestingly, in a human cuspid distalization model, pro-inflammatory cytokine (interleukin-1β, monocyte chemoattractant protein-1, tumor necrosis factor-α) and osteoclast markers (RANKL and matrix metalloproteinase-9) in the gingival crevicular fluid were higher in adults than adolescents at 1, 7 and 14 days after application of orthodontic forces, albeit OTM was slower in adults^23^. In our present study, the labial ABA in the incisor area was increased by 2.13 ± 2.58 mm^2^ and 1.84 ± 2.47 mm^2^ for the central and lateral respectively in the adolescent group, while the increase was only 1.16 ± 2.55 mm^2^ and 0.73 ± 2.38 mm^2^ in the adult group. Therefore, the age-related biological features in osteoblasts underline the less bone formation in adults after orthodontic treatment. Less alveolar bone was observed in the adult subjects in our present study, indicating a potential periodontal risk on a long-term basis.

To minimize the influence of distance of incisor retraction on alveolar bone, we only included patients who need moderate anchorage while excluded patients who need maximal anchorage. We may presume a similar amount of tooth movement in both groups in our present study, since the incisor inclination in both groups were similar before and after treatment. However, it must be noted that the buccal/lingual inclination, also called torque, is determined majorly by the ratio of momentum/force (M/F)^30^. Similar inclination of upper incisors cannot exclude bodily movement during retraction. Since a greater tendency to anchorage loss in adolescents was observed than in adults^31,32^, larger amount of retraction might be anticipated in adult subjects. The larger anchorage loss in adolescents might be attributed to more robust biological response to similar orthodontic force (mechanical anchorage loss) or molar drifting after extraction during growth (physiologic anchorage loss)^33^. Therefore, larger ABA in adolescents may still be a consequence of less incisor retraction.

We excluded the influence the sex and ethnicity by recruiting girls younger than 16 years old in the adolescent group, and females older than 18 years to minimize influence of growth in the adult group. In a Caucasian population, lateral cephalograms were obtained biennially between the ages of 4.5 and 12 years and annually through age 17, and finally at the age of 25, finding that boys have a later growth pattern after the age of 17 in the mandibular region with far more changes in the S–N-Pog°, Pog-NB(mm) and Ar-Pog(mm)^34^; similarly, mandibular growth was greater in the men from late adolescence (mean age, about 17 years) than midadulthood (mean age, about 47 years)^35^. In addition, Information regarding the longitudinal growth of alveolar bone in adolescents is not available in the Chinese population; however, height spurt peak age was earlier in the girls (10.98 ± 0.95 years) than boys (12.72 ± 0.89 years)^36^; therefore, we selected the female subjects in the study to reduce the confounding of growth on the alveolar bone remodeling.

We excluded cases with history of periodontal diseases, since periodontitis history would impact the onset of periodontal inflammation by the genetic and epigenetic backgound^37^. Although we observed favorable alveolar bone support on the palatal side in the adolescent group, significant alveolar bone loss might still be a problem in the adolescent when maximal anchorage is applied.

The retrospective nature of the present retrospective study may influence our explanation of the data. Apart from the age-related orthodontic events may affect alveolar bone remodeling, periodontal disease history, oral hygiene motivation, sex hormone and medication may significantly impact periodontal status during orthodontic treatment^38^. Moreover, one drawback is that the data for the clinical periodontal status, including gingival recession, clinical attachment level and bleeding on probing, were not available in this retrospective study. The height and thickness of alveolar bone are critical factors to protect the teeth from the gingival recession^39^, and OTM in adults may enhance the prevalence of periodontal dehiscences and fenestrations^40^; therefore, clinicians should not be blinded to the periodontal risks induced by mass retraction in adult patients. Further longitudinal studies are needed to explore whether the reduction in the alveolar bone support in the adult group may really lead to the gingival recession.

In our present study, we utilized alveolar bone distance, alveolar bone thickness and alveolar bone area to reflect the alveolar bone support after OTM. All these parameters consistently and clearly demonstrated that adolescents showed favorable alveolar bone support after treatment. Therefore, clinicians should pay attention to the application of orthodontic force during incisor retraction, and perform radiological examinations on patients with a high risk of periodontal bone loss. Moreover, alveolar bone undergoes dynamic remodeling even after orthodontic treatment. In one case report, significant alveolar bone formation was observed on the palatal side of the maxillary incisors 10 years after retention^41^. Most recently, Wang et. al. reported that bone apposition is observed 18 ~ 24 month after incisor retraction in adolescent patients^20^. Further longitudinal study should be conducted to explore the difference of alveolar bone support between the adolescents and adults in the long term.

## Conclusions

On the basis of the evidence currently available, the findings of this retrospective study are summarized as follows:

1. More alveolar bone loss was observed in adult patients, and age may become a risk of alveolar bone resorption during orthodontic treatment.
2. Alveolar bone remodeling displayed a with-the-bone pattern in adolescents, while a through-the-bone pattern in adults.
3. Age should be taken into consideration during orthodontic treatment planning to reduce periodontal risk.

## Data Availability

The datasets used and/or analyzed during the current study are available from the corresponding author on reasonable request.
